# Extraction of Active Compounds from Mixtures of Hemp (*Cannabis sativa*) with Plants of the *Zingiberaceae* Family

**DOI:** 10.3390/molecules28237826

**Published:** 2023-11-28

**Authors:** Vesna Postružnik, Taja Žitek Makoter, Darko Goričanec, Petra Kotnik, Željko Knez, Maša Knez Marevci

**Affiliations:** 1 Laboratory for Separation Processes and Product Design, Faculty of Chemistry and Chemical Engineering, University of Maribor, 2000 Maribor, Sloveniataja.zitek@um.si (T.Ž.M.); petra.kotnik@um.si (P.K.); zeljko.knez@um.si (Ž.K.); 2 Laboratory of Thermoenergetics, Faculty of Chemistry and Chemical Engineering, University of Maribor, 2000 Maribor, Slovenia; darko.goricanec@um.si; 3 Faculty of Medicine, University of Maribor, Taborska 8, 2000 Maribor, Slovenia

**Keywords:** supercritical fluid extraction, ultrasound-assisted extraction, hemp, ginger, turmeric, cardamom

## Abstract

Hemp is probably one of the most studied plants for its health-promoting properties, with countless documented and patented extraction methods, but literature is scarce on the simultaneous extraction of mixture of raw materials. Hemp, along with other plant materials, could represent a potentially highly valuable source material with resulting reciprocal effects. In this study, hemp (*Cannabis sativa*) and three members of the *Zingiberaceae* family, ginger (*Zingiber officinale*), turmeric (*Curcuma longa*), and cardamom (*Elettaria cardamomum*), were extracted simultaneously, and their bioactive component values were investigated. Two extraction methods were used, namely ultrasound-assisted extraction with ethanol and supercritical fluid extraction with carbon dioxide. First, extracts were obtained from separate plant materials. Then, hemp was extracted in combination with ginger, turmeric, and cardamom in a 1:1 ratio. The extracts obtained were evaluated for their antioxidant activity and total phenolic content using UV/VIS spectrophotometry; cannabinoid content, 6-gingerol, and 6-shogaol were measured using liquid chromatography coupled with tandem mass spectrometry (LC-MS/MS); volatile components such as 1,8-cineole, alpha-terpinyl acetate, linalool, and aR-turmerone were measured using gas chromatography with mass spectrometry (GC/MS).

## 1. Introduction

Combinations of herbs have been used to treat illnesses for thousands of years, and even in modern pharmacy, a large fraction of remedies are still prepared directly from natural materials. Healthy lifestyles and nutrition are becoming increasingly important concepts, which is why the tendency to use natural products to replace synthetically produced compounds and dietary supplements has increased recently. Furthermore, the list of plants used in herbalism with confirmed medicinal efficacy is growing longer and longer. Nevertheless, few studies have been published that investigate mixtures of natural extracts. In this study, four different medicinal plant materials with well-documented beneficial health properties were used to prepare natural extracts with high levels of biologically active ingredients. These were hemp (*Cannabis sativa*) and three representatives of the *Zingiberaceae* family: ginger (*Zingiber officinale*), turmeric (*Curcuma Longa*), and cardamom (*Elettaria cardamomum*). In addition, a suitable extraction method, solvent, and combination of plants with synergistic effects can improve the quality of the product [1]. Hemp extracts have been shown to have therapeutic properties, most of which are associated with cannabinoids and especially cannabidiol (CBD) [2]. The three plants from the *Zingiberaceae* family with similar chemical compositions have high levels of phenols and free radical scavengers that reduce inflammation, cardiovascular diseases, and some cancers. In our previous work we have already indicated that hemp has synergistic effects when combined with ginger, increasing the overall yield of cannabidiol when extracted in combination [3,4]. This study further examined the synergistic effects of turmeric and cardamom compounds. In the last decade, the concept of extraction of plant mixtures with supercritical CO_2_ has been investigated by several authors, and synergistic effects between plant combinations have been confirmed. Ivanovic et al. confirmed an increase in extraction rate between clove bud and oregano and clove bud and thyme mixtures, leading to an increase of up to 90% of the extraction yield compared to extraction of pure clove [5]. The authors state that compounds present in oregano and thyme could act as modifiers or co-solvents and therefore change the CO_2_ solubility power. Furthermore, the plant combinations showed synergistic effects in terms of antibacterial and antioxidant activities [5]. Maksimovic et al. studied supercritical fluid extraction with CO_2_ of sage leaves and curry flower mixtures, which resulted in a significant increase in monoterpenes compared with extraction of individual plant materials. The authors attributed this effect to the co-solvent effect of sage on specific monoterpenes, sesquiterpenes, and diterpenes, which are abundant but caged in curry. Extraction of plant mixtures was found to increase the amount of heavier compounds from curry flower, which are actually not present in the pure essential oil of sage [6].

Since herbal remedies have multi-layered interactions in the treatment of diseases, they resemble the effects of several synthetic drugs with multiple targets [7]. When herbal combinations are used, various interactions may occur between individual ingredients that should provide better efficacy than equivalent doses of individual herbs. The most desirable interactions provide additional therapeutic benefits or reduce side effects, with the components working together in the body to maintain health or combat disease, meaning that a greater overall effect can be achieved than with the individual active ingredients.

Mixtures of biologically active compounds from natural materials, their extracts, or essential oils may increase antioxidant capacity to inhibit oxidative processes or antimicrobial and anti-inflammatory activity in the human body, or produce other effects [8]. Adverse effects should also be closely monitored, as they may also be enhanced when plant combinations are used. The mechanisms responsible for various biological activities, especially antioxidant activity, are still poorly understood due to the complex composition of plant extracts. Considering all aspects of how substances enter and move in the body and how they are excreted helps us to assess the changes of active substances in the body and their therapeutic effects.

To our knowledge, the literature on the extraction of combinations of hemp and plants of the Zingiberaceae family is scarce, and no data of their synergistic effect can be found. In our previous work, we confirmed the positive medicinal effects of hemp–ginger combinations on the metabolic activity of metastatic cells and against microorganisms [3]. The aim of this study was to continue this research and explore the interactions that occur during the extraction process and the levels of chemical compounds responsible for some of the medicinal effects of these plants. Cardamom and turmeric were also included in the study as they belong to the same plant family and have well-documented health benefits similar to ginger. So far, no evidence of toxicity of the plant mixtures has been found. All plants involved are used as spices and are generally considered safe for use in food, and many recipes for various foods can be found online that include all the combinations used in this study.

## 2. Results and Discussion

The plant extracts from the materials used in this study have well-documented antioxidant activity and high phenolic compound content [9,10,11,12,13], but this study is the first to evaluate the mutual effects of plant combinations on their content. In addition, the biologically active components of each plant were reviewed and selected for evaluation of their potential synergistic effects during extraction. The therapeutic efficacy of hemp has been reported to be due to cannabinoids [14,15,16]. Therefore, seven of them, including cannabidiol, were evaluated for hemp-containing extracts. For extracts containing ginger, the main active ingredients were determined to be 6-gingerol and 6-shogaol [17]. The main active ingredient of turmeric essential oil, aR-turmerone [18] was evaluated, while for extracts containing cardamom, the main bioactive components of the essential oil, alpha-terpinyl acetate, 1,8-cineole, and linalool [19] were evaluated.

### 2.1. Antioxidant Activity and Total Phenolic Content

**Figure 1:**
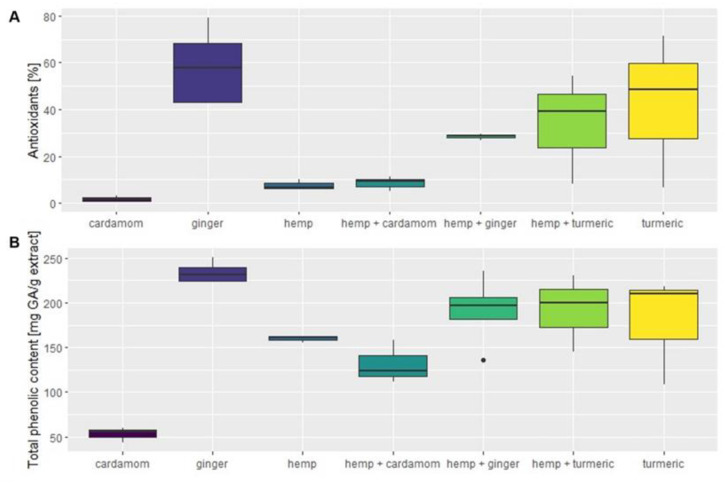
(**A**) Antioxidant activity [%]. (**B**) Total phenolic content [mg GA/g extract] of the plant extracts according to the material and their mixtures.

Boxplots of antioxidant activity and total phenolic content depending on the plant material, marked with separate colors, are shown in Figure 1A,B and Appendix A respectively, showing the distribution of data for extraction replicates. A five-digit summary is shown for each material, including the minimum and maximum values at the outer ends of the plot, the first and third quantiles at the two box edges, and the medians in between. Figure 1 was created with the following program sequence:

p1 < -ggplot (df, aes (x = Material, y = Antioxidants, fill = Material)) +

geom_boxplot ()+

labs (y = “Antioxidants [%]”,x = element_blank ())+

theme (legend.position = “none”)+

scale_fill_viridis (discrete = TRUE)

p2 < -ggplot (df, aes (x = Material, y = Total_Phenols, fill = Material)) +

geom_boxplot()+

labs (y = “Total phenolic content [mg GA/g extract]”, x = element_blank ())+

theme (legend.position = “none”)+

scale_fill_viridis (discrete = TRUE)

ggarrange (p1, p2,

labels = c (“A”, “B”),

nrow = 2)

Depending on the plant material, the antioxidant activity varied significantly, which was also confirmed with the Kruskal–Wallis statistical test (F(6) = 14.372; *p* < 0.026). Figure 1A shows the values of antioxidant activity depending on the plant material, while the corresponding data are also listed in the Table A1 in Appendix A. Ginger and turmeric had the highest median antioxidant activity of 57.69 (±17.98)% and 48.38 (±32.48)%, respectively, while hemp and cardamom had the lowest values of 6.64 (±2.13)% and 1.74 (±1.35)%, respectively. The antioxidant activity of the plant mixtures fell between these values for hemp and *Zingiberaceae* mixtures, except for cardamom, whose mixture with hemp had higher radical scavenging activity than other plant materials extracted separately. Hemp–cardamom mixtures had a median antioxidant activity of 9.23 (±3.12)%, but post hoc Dunn test did not confirm significant differences between the hemp–cardamom mixture and separate plant materials (*p* > 0.05). Nevertheless, ultrasonic-assisted extraction (UAE) showed higher values for antioxidant activity for the hemp–cardamom mixture than for the separate materials. Repetition of measurements would be necessary for statistical confirmation of the synergistic effect of these two plants separately. A synergistic effect was also observed with the supercritical extraction of the mixture of industrial hemp and turmeric, which achieved a higher percentage of inhibition than the individual materials. Comparison of extraction methods showed that UAE gave higher antioxidant yields than SCE for most materials, with the exception of ginger and cardamom, where the opposite was true. Figure 1 shows broader interquartile ranges for ginger, turmeric, and hemp–turmeric, which are attributed to the differences in extraction methods. The median antioxidant activity of turmeric was 71.51 (±6.08)% for UAE extraction, while SCE achieved only 6.56 (±0.49)%. Similarly, for the hemp–turmeric mixture, the UAE method was again more efficient, with a median antioxidant capacity of 54.23 (±4.56)%, while the median value of SCE was 8.23 (±0.32)%. In contrast, the median antioxidant activity of ginger was higher with SCE, with the highest median antioxidant activity of 79.17 (±4.67)%, while the UAE extraction had a median antioxidant activity of 43.2 (±3.02)%. Thus, the effectiveness of the extraction method for antioxidant activity is highly dependent on the plant material. Since more polar components are extracted by UAE and more nonpolar components are extracted by SCE, we can claim that many polar and nonpolar components are present in the analyzed samples.

In Figure 1B, total phenolic content in mg GA per g extract is shown for plant materials and their mixtures. The corresponding data are also listed in the Table A1 in Appendix A. The values follow a similar distribution to the antioxidant activity, while significant differences between natural materials and their mixtures were also confirmed with ANOVA (F(6) = 8.028; *p* < 0.001). Like the antioxidant activity, ginger had the highest average total phenolic content of 237.96 (±13.2) mg GA/g extract, followed by the mixture of hemp–turmeric with 191.88 (±43.07) mg GA/g extract, the mixture of hemp–ginger with 189.20 (±50.00) mg GA/g extract, and turmeric with 179.14 (±61.32) mg GA/g extract. The value of total phenolic content was lowest in cardamom, with 52.93 (±8.35) mg GA/g extract, while hemp showed a comparatively higher extraction efficiency of total phenolic components of 159.84 (±3.74) mg GA/g extract. Synergistic effects were obtained via UAE for industrial hemp–turmeric mixtures and industrial hemp–ginger mixtures, as the values of total phenolics obtained in the above extractions were higher than in the extracts of the individual materials. UAE gave the highest mean of total phenolic content for all hemp and *Zingiberaceae* mixtures. Smaller interquartile ranges can be seen on Figure 1B compared to Figure 1A, but again, turmeric and hemp–turmeric show the greatest differences in extraction methods. The UAE for turmeric had nearly twice the values of SCE, with a mean of 210.67 (±3.58) mg GA/g extract compared to 108.47 (±13.02) mg GA/g extract.

With natural extracts, variations in observed content of bioactive molecules is expected and a consequence of differences in material preservation, harvest period, climatic, geomorphological conditions, etc. [20,21,22]. Nevertheless, the measured results of antioxidant activity and total phenolic content concur well with the literature, and a broad spectrum of bioactive components with antioxidant properties is characteristic for these plants, as well as high values of phenolic components [9,10,11,12,13]. The cardamom total phenolics, which were the lowest among the materials measured, aligns well with results that Oueslati et al. obtained from ethanolic extracts, 51.7 mg GA/g extract [23], and are even higher than results from ethanolic extracts that Tarfaoui et al. obtained, 33.45 (±0.35) mg GA/g extract [12]. Furthermore, the values for ginger and turmeric, with the highest TPC difference between materials, are similar to those reported by Sepahpour et al. for turmeric ethanolic extracts, 172.1 (±1.4) mg GA/g extract [24], and by El-Sayed for ginger methanolic extract, 266.7 mg GA/g extract [25].

### 2.2. Cannabinoid Content

**Figure 2:**
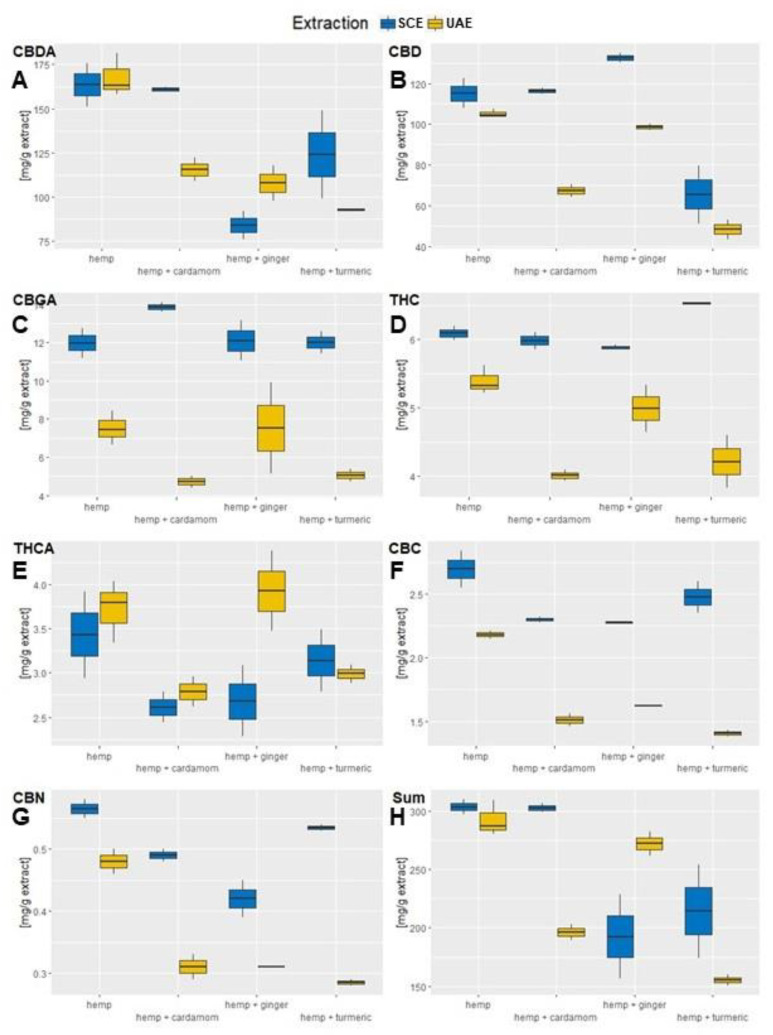
Comparison of cannabinoid content between SCF and US extraction. (**A**) The content of mg CBDA per g extract. (**B**) The content of mg CBD per g extract. (**C**) The content of mg CBGA per g extract. (**D**) The content of mg THCA per g extract. (**E**) The content of mg CBC per g extract. (**F**) The content of mg CBN per g extract. (**G**) The content of mg of total measured cannabinoids per g of extract. (**H**) The content of mg Cannabinoids (CBDA, CBD, CBGA, THCA, CBC, CBN) per g extract.

In this study, seven cannabinoids were measured, namely Cannabidiolic acid (CBDA), Cannabidiol (CBD), Cannabigerolic acid (CBGA), Δ9–tetrahydrocannabinol (THC), Δ9-tetrahydrocannabinolic acid (THCA), Cannabichromene (CBC), and Cannabinol (CBN), of which the most abundant were CBDA, followed by CBD, as shown in Figure 2 and Table A2 in the Appendix A. The extraction of bioactive components of industrial hemp in the case of CBDA had highest values for industrial hemp as a single material, since in this case the majority was extracted, i.e., 160.07 (±18.3) mg CBDA/g extract. The most effective mixture was obtained in the interaction with cardamom, with 147.9 (±21.8) mg/g extract. Both extraction methods had similar CBDA values.

What is interesting about CBD is that the hemp–cardamon mixture (109.26 ± 20.89 mg CBD/g extract) exceeded the value of industrial hemp alone (107.64 ± 9.06 mg CBD/g extract). Although the values between materials are not significantly different, a strong synergistic effect of ginger on CBD for all replicates can be observed. Furthermore, the mixture of industrial hemp with cardamom approaches the CBD value of industrial hemp. Such behavior is achieved only for SCE of the industrial hemp–ginger mixture, where more CBD components were extracted from the mixture than from industrial hemp itself. Only the mixture of hemp–turmeric did not show a positive effect in any case.

The third most abundant cannabinoid in the hemp extracts was CBGA. The two-way ANOVA confirmed the influence of the extraction method (F = 22.05, *p* > 0.001) on the CBGA content but not for material mixtures (F = 1.33, *p* = 0.31). The SCE extraction was the most efficient method for CBGA extraction, and for this method, all hemp mixtures achieved higher CBGA values than hemp alone, but this was not the case for UAE, where only the hemp–ginger mixture surpassed the sole material. The highest extraction efficiency of CBGA was achieved for SCE of the hemp–cardamom mixture, with the highest measurement of 14.13 mg CBGA/g extract, while hemp’s highest measurement was 12.76 mg CBGA/g extract.

For THC, the two-way ANOVA showed a significant influence of the extraction method (*p* < 0.001), but not of the material (*p* = 0.31). SCE was most efficient for THC extraction for all materials, while the values of THC for SCE of hemp–turmeric exceeded the values of hemp as a single component but not for UAE. The same pattern was observed for THCA, where extraction method significantly influenced the obtained values (*p* = 0.04); however, the combinations of materials used did not (*p* = 0.07). Contrary to THC, UAE was more effective for the extraction of THCA. UAE of hemp–ginger produced the highest content of THCA, with 3.93 (± 0.64) mg THCA/g extract, even higher that UAE extraction of hemp alone, with 3.72 (±0.35) mg/g extract, yet not significantly different.

CBC and CBN values were present in minor amounts, and for both components, SCE reached significantly higher values compared to UAE (*p* < 0.05). Hemp as a separate material had the highest component content, followed by the hemp–turmeric mixture. Nevertheless, the latter also had the biggest difference in extraction method efficiency, as UAE extraction produced lower quantities of CBC and CBD than any other mixture.

Additionally, the sum of all cannabinoids was calculated, where SCE of sole hemp proved to be the most effective method, reaching 303.43 mg/g extract. However, hemp–cardamom SCE reached almost the same levels, with 302.60 mg/g extract. It is important to state that plants of the *Zingiberaceae* family do not contain any cannabinoids, and as hemp mixtures contained 50% less hemp material than the individual material, the cannabinoid content without any synergistic effects should therefore be 50% lower than in the mixtures, which is clearly not the case. Instead, the cannabinoid content in mixtures is comparable and, in several cases, even exceeds the hemp values extracted separately. Comparing to the literature, optimized SCE methods reached up to 305.8 mg CBGA/g extract for cannabis inflorescences [26], which is roughly twice the levels achieved in our extraction of hemp inflorescences and leaves. Furthermore, extractions from cannabis residues were directly comparable to our results in CBDA levels, which were between 157.6 (±6.0) mg CBDA/g extract and 261.4 (±2.2) mg CBDA/g extract, with lower CBD values between 18.28 (±0.68) mg CBD/g extract and 64.18 ± 2.98 mg CBD/g extract [27]. Similar results were found for CBD in medicinal cannabis SCE plant material extracts, with values between 103.2 mg CBD/g extract and 191.7 mg CBD/g extract, while THC values were, in this case, considerably higher, reaching 761.1 mg THC/g extract [28].

### 2.3. Other Bioactive Components

**Figure 3:**
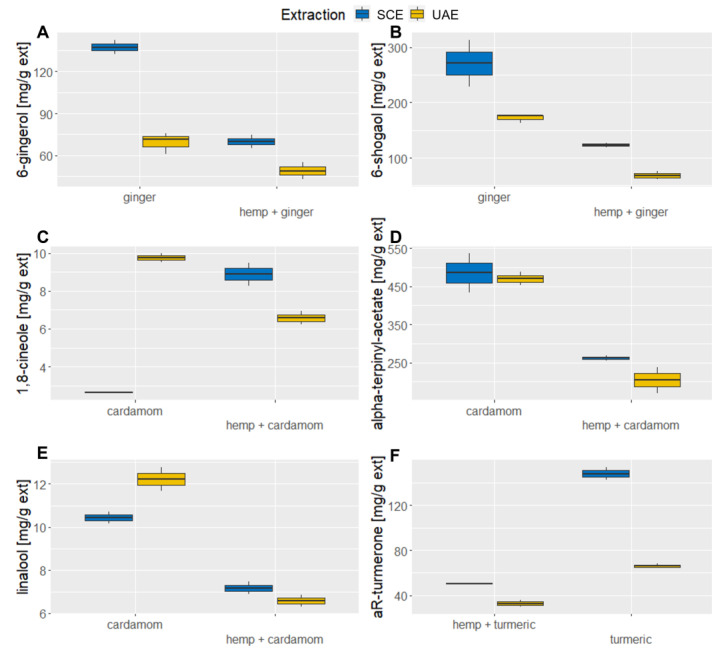
Comparison of biologically active components between materials and extraction methods. (**A**) The content of 6-gingerol in ginger and hemp–ginger mixture. (**B**) The content of 6-shogaol in ginger and hemp–ginger mixture. (**C**) The content of 1,8-cineole in cardamom and hemp–cardamom mixture. (**D**) The content of alpha-terpinyl acetate in cardamom and hemp–cardamom mixture. (**E**) The content of linalool in cardamom and hemp–cardamom mixture. (**F**) The content of aromatic-turmerone in turmeric and hemp–turmeric mixture.

The obtained results, shown in Figure 3A,B with detailed information in Table A3 in the Appendix A, show that the contents of both 6-gingerol and 6-shogaol are significantly higher in ginger alone than in its mixture with industrial hemp. The addition of industrial hemp does not lead to higher extraction efficiency. In the case of 6-gingerol, its value in the mixture is reduced by a factor of one compared to ginger alone, while in the case of 6-shogaol, this value increases to about three times. 6-gingerol was isolated to the greatest extent during SCE of ginger alone (137.38 ± 7.21 μg/mg extract), followed by SCE of hemp–ginger (69.98 ± 6.39 mg/g extract). A similar trend of results can also be observed with 6-shogaol, where SCE of ginger also proved to be the most effective (271.12 ± 59.93 mg/g extract), followed by UAE of sole ginger (172.25 ± 8.24 mg/g extract).

It is important to note that hemp as an individual material does not contain 6-shogaol and 6-gingerol; therefore, without any synergistic effects during extraction, half of the amounts of both components are expected, which is the case for 6-gingerol. For 6-shogaol, the values in material mixtures were even lower, indicating that a negative synergistic effect has occurred. For ginger SCE extracts, Kamaruddin et al. reported 6-gingerol values between 72.19 mg/g extract and 167.78 mg/g extract [29], while for UAE ethanolic extracts, lower values were reported by Ok and Jeong (25 mg/g extracts of 6-gingerol and 57 mg/g of 6-shogaol) [30].

Figure 3C shows 1,8-cineole, which is present in cardamom. The corresponding data are presented on Table A4 in Appendix A. Using UAE, it was extracted in the highest amounts, but hemp mixtures far exceeded the half values and, in case of SCE, far exceeded the individual material, which indicates a potent interaction between materials. However, this cannot be said for alpha-terpinyl acetate and linalool, where the mentioned components are extracted almost twice as much from cardamom as from the hemp mixture.

Considering the influence of the extraction method on the secretion of 1,8-cineole, the largest quantity of it was obtained via UAE of cardamom (9.74 ± 0.33 mg/g extract), and slightly less through SCE of hemp–cardamom (8.89 ± 0.86 mg/g extract), while SCE of cardamom turned out to be the least efficient method (2.65 ± 0.06 mg/g extract). These results are supported by the solubility of biologically active components, which in our case means that 1,8-cineole is a more polar component and is extracted via UAE. In the case of alpha- terpinyl acetate, shown in Figure 3D, as a non-polar component, the situation is exactly the opposite. Most of the mentioned component was extracted precisely during the SCE of cardamom (485.54 ± 71.82 mg/g extract). As linalool is highly soluble in ethanol, the UAE of pure cardamom was the most efficient (12.23 ± 0.78 mg/g extract), as shown in Figure 3E. The hemp mixture reached higher values than half for linalool (7.18 ± 0.4 mg/g extract). The SCE values were lower but comparable for individual material (10.44 ± 0.4 mg/g extract) and mixtures (6.58 ± 0.28 mg/g extract).

Paul and Bhattacharjee reported that SCE extract contained between 11.93 mg of 1,8-cineole per g dry seeds and 32.18 mg/g dry seeds [31], which is comparatively higher than the results reported in this study. Furthermore, Morsy reported 1,8-cineole values in essential oils after different treatments that were between 10.93 mg/mL and 13.31 mg/mL, as well as alpha-terpinyl acetate levels between 8.20 mg/mL and 14.50 mg/mL [32]. To our knowledge, no studies have been published reporting the mass of 1,8-cineole, alpha-terpinyl acetate, and linalool in cardamom per mass of extract.

Figure 3F shows the content of aR-turmerone in extracts, while the corresponding data are also listed in the Table A5 in Appendix A. The results indicate a higher efficiency of turmerone secretion in turmeric SCE extract (147.93 ± 8.15 mg/g extract). This value decreases to 50.73 ± 0.52 mg/g extract in the mixture of hemp–turmeric. Using UAE, the values are even lower. Like ginger, turmeric works best as a stand-alone ingredient. The extraction methods used showed the highest efficiency of turmerone extraction in SCE of turmeric (147.93 ± 8.15 mg/g extract). UAE followed with the second highest values (66.01 ± 2.09 mg/g extract), where turmeric was extracted without the addition of industrial hemp, and the mixtures had lower values for both extraction methods. Kongpol et al. report 2.80 (±0.29) wt%/dry basis aR-turmerone for turmeric UAE ethanolic extracts [33], while Carvalho et al. reported a yield of aR-turmerone of up to 1.14% per raw material for SCE turmeric extracts [34]. However, we have again found no data on the aR-turmerone mass per mass of extract.

### 2.4. Correlation Analysis

**Figure 4:**
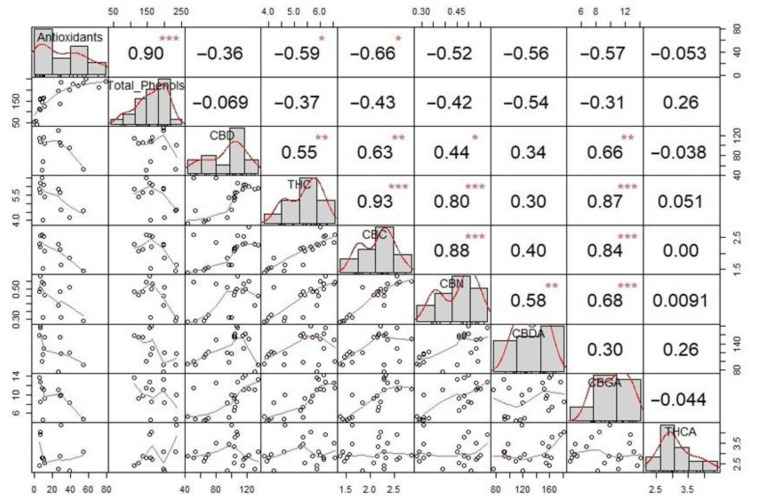
Correlation analysis for antioxidant activity, total phenolic content, and cannabinoids in hemp extracts and their mixtures. Significance levels *—*p* < = 0.05, **—*p* < = 0.01, ***—*p* < = 0.001 are displayed above Spearman’s correlation coefficients.

Correlation analysis was conducted using Spearman’s correlation tests. In Figure 4, the correlation matrix for cannabinoids is shown. Plot distribution is shown on the plot diagonal, above which the values of Spearman’s correlation test are displayed, with significance levels (* *p* < = 0.05, ** *p* < = 0.01, *** *p* < =0.001), and below which are bivariate scatter plots with a fitted line. A significant strong linear correlation (r = 0.90, *p* < 0.001) was found between antioxidant activity and total phenolic content. Phenolic compounds are considered the most important antioxidants and are widespread among different plant species, so for this purpose, the correlation of antioxidants with the total content of phenols was expected and confirmed for hemp, ginger, and turmeric in previous studies [35,36]. The high correlation value confirms the role of phenolic compounds as the main factor contributing to the antioxidant activity of the extracts of the analyzed materials. Moderate linear correlation to antioxidant activity was also found for most cannabinoids except for CBD, CBN, and THCA. Also, moderate to strong correlations were found for 11 out of 21 groups of cannabinoids. However, no significant correlations were found between cannabinoids and total phenolic content, which indicates that the analyzed extracts contain other non-cannabinoid phenols such as flavonoids, terpenes, and alkaloids [37].

**Figure 5:**
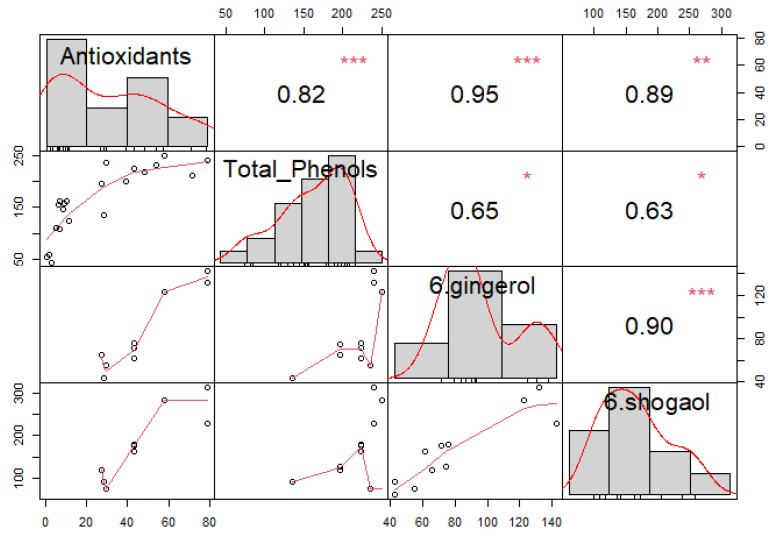
Correlation analysis for antioxidant activity, total phenolic content, 6-gingerol, and 6-shogaol in hemp extracts and their mixtures. Significance levels *—*p* < = 0.05, **—*p* < = 0.01, ***—*p* < = 0.001 are displayed above Spearman’s correlation coefficients.

For materials containing ginger, correlation analysis between antioxidants, total phenols, 6-gingerol, and 6-shogaol was conducted. Moderate to strong significant correlations were determined for all components, as presented in Figure 5. Histograms are displayed on the diagonal, above which are Spearman’s correlation coefficients with significance levels (* *p* < = 0.05, ** *p* < = 0.01, *** *p* < = 0.001) and below which are bivariate scatter plots with a fitted line. Even though both cases achieve a positive correlation coefficient, a stronger correlation is achieved in the case of the dependence of antioxidants on 6-shogaol, (r = 0.89, *p* < 0.01) for AA and (r = 0.7, *p* < 0.05) for TPC, compared to (r = 0.86, *p* < 0.01) for AA and (r = 0.67, *p* < 0.05) for TPC. This was already confirmed by Guo et al. (2014), who also observed that the antioxidant capacity of 6-shogaol was greater than that of 6-gingerol. The reason for this is attributed to the presence of α,ꞵ-unsaturated ketone parts of 6-shogaol [38]. It should also be mentioned that statistical analyses were performed for 8-gingerol and 8-shogaol and 10-gingerol and 10-shogaol, but no meaningful results were obtained, which we attribute to the length of the carbon chains. The shorter the carbon chain of ginger’s biologically active components, the stronger its antioxidant properties. Therefore, we can say that 6-shogaol and 6-gingerol are some of the main components responsible for the antioxidant activity [38].

**Figure 6:**
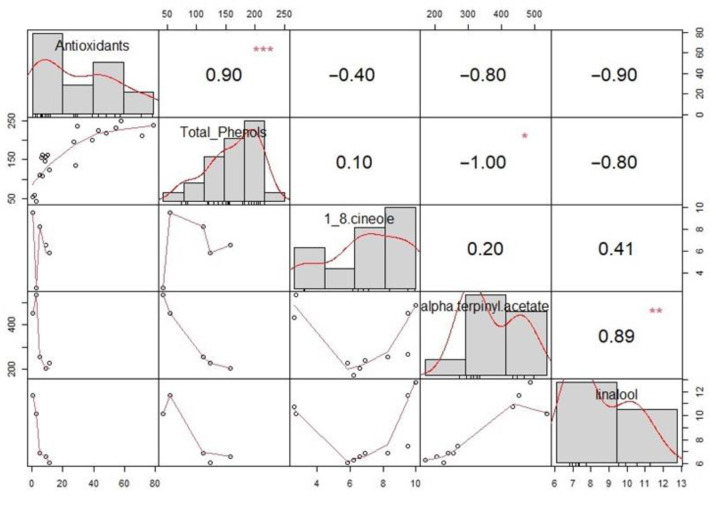
Correlation analysis for antioxidant activity, total phenolic content, 1,8-cineole, alpha-terpinyl acetate, and linalool. Significance levels *—*p* < = 0.05, **—*p* < = 0.01, ***—*p* < = 0.001 are displayed above Spearman’s correlation coefficients.

For extracts containing cardamom, correlation analysis of antioxidant activity, total phenolic content, and 1,8-cineole, alpha-terpinyl acetate, and linalool content was performed. Spearman’s correlation tests were performed for variables and are visualized on the correlation matrix—Figure 6. On the diagonal, a histogram of each plot is shown, above which are the diagonal Spearman’s correlation values along with the significance levels (* *p* < = 0.05, ** *p* < = 0.01, *** *p* < = 0.001) and below which the diagonal scatter plots with a fitted line are positioned. Alpha-terpinyl acetate showed a lower antioxidant activity, a negative but not significant correlation to the latter, and a strong inverse correlation with total phenolic content. The data obtained is consistent with the literature [39], stating that the low antioxidant and radical scavenging activity of alpha-terpinyl acetate, a menthane monoterpene, occurs due to its chemical nature. Furthermore, 1,8-cineole and linalool did not show a significant correlation between antioxidant activity and total phenolic content. In general, for cardamom, the lowest values of these two parameters were characteristic among all plant materials tested. A correlation was confirmed between linalool and alpha-terpinyl acetate (r = 0.89, *p* < 0.01), indicating that these components are extracted in direct proportion for both extraction methods.

## 3. Materials and Methods

This study focused on evaluating the content of selected chemical components when combinations of plants are extracted in mixtures and aims to quantify their content in extract combinations compared to single materials. The objective was to investigate the potential synergistic effects of mixtures of industrial hemp with ginger, turmeric, and cardamom. To this end, we first obtained extracts from single plant materials and then used mixtures of plant materials for comparison. Industrial hemp was used in combination with ginger, turmeric, and cardamom in a 1:1 ratio. The three members of the *Zingiberaceae* family were selected for their antioxidant, anti-inflammatory, and other healing properties. Ultrasonic–assisted extraction with food-grade ethanol was chosen for the extraction of the polar components, while supercritical CO_2_ was selected for the extraction of the nonpolar components. The latter enables a green, environmentally sustainable process that does not use organic solvents or other chemicals and yields a pure extract. Several studies have already been published on the extraction of these compounds, but our goal was to achieve a higher yield of high-value components such as cannabinoids, 6-gingerol, 6-shogaol, aromatic turmerone, alpha-terpinyl acetate, linalool, and 1,8-cineole.

The dried natural materials of the *Zingiberaceae* family (ginger rhizome, turmeric rhizome, and cardamom seeds) used in the study were obtained from Alfred Galke, while industrial hemp inflorescences and leaves were obtained from a local organic farmer. The natural materials were stored at room temperature in a dry, dark environment. Before starting the extraction process, all materials were ground to a particle size of 150 µm. Based on the literature, two optimized extraction methods were selected for the critical separation step: ultrasonic and supercritical extraction [2,40,41]. In the study, natural materials were extracted individually and in combinations. Each *Zingiberaceae* family natural compound was mixed with hemp in a 1:1 ratio.

### 3.1. Ultrasonic Assisted Extraction with Ethanol

Ultrasonic-assisted extraction (UAE) is a technique for isolating bioactive compounds from a plant, which has applications in the food and pharmaceutical industries, among others. The energy of ultrasonic waves in the solvent causes cavitation, accelerating dissolution, diffusion of solutes, and heat transfer, positively affecting extraction efficiency. Ultrasonic treatment of each material was carried out for 30 min at a room temperature of 25 °C. The obtained mixture was filtered, and the solvent was removed under a vacuum at 40 °C. The procedure for obtaining the extract was repeated three times, and the obtained extracts were stored at −20 °C until analysis.

### 3.2. Supercritical Fluid Extraction with Carbon Dioxide

Supercritical fluid extractions (SCE) of natural products were performed according to the procedure as described by Žitek et al. [3]. The system was preheated and then maintained at 50 °C. The autoclave was filled with 25 g of natural ground material or a 1:1 mixture of materials. During the extraction process, the supercritical carbon dioxide (solvent to feet ratio 8.3) was uniformly introduced into the autoclave at a pressure of 250 bar. The supercritical fluid flowed through the material and dissolved the soluble components, which were then collected in a pre-weighed test tube. The procedure for obtaining the extract was repeated three times, and the obtained extracts were stored at −20 °C until analysis.

### 3.3. Spectrophotometric Analyses

Spectrophotometric measurements were performed for examination of the extracts on their antioxidant activity and total phenolic content.

### 3.4. Determination of Antioxidant Activity

Extract antioxidant activity (AA) was determined using the 1,1-diphenyl-2-picrylhydrazyl (DPPH) reagent as described by Huang et al. [42]. Briefly, 10 mg of extract was dissolved in 10 mL of methanol. The solution was dissolved using an ultrasonic bath. A total of 3 mL of the DPPH solution and 77 μL of the extract solution were mixed and thermostated for 15 min at room temperature before the absorbance at 515 nm was measured.

### 3.5. Determination of Total Phenolic Content

The total phenolic content (TPC) was measured using the Folin–Ciocateu (FC) reagent, as described by Škerget et al. [43]. Briefly, 40 mg of extract was diluted in 20 mL of distilled water and added to 2.5 mL FC reagent diluted 10-fold with distilled water, and 2 mL Na_2_CO_3_ (75 g/L) was added. Distilled water was used as the control. Using gallic acid (GA), the calibration curve was prepared. The glass vials were thermostated at 50 °C for 5 min, cooled, and the absorbance was measured at 760 nm. The total phenolic content was expressed as mg GA per g of extract.

### 3.6. Chromatographic Analyses

Liquid chromatography coupled with tandem mass spectrometry (LC-MS/MS) and gas chromatography with mass spectrometry (GC/MS) were used to measure biologically active components in plant extracts. Representative chromatograms are shown in the Supplementary Materials.

### 3.7. Liquid Chromatography with Mass Spectrometry (LC-MS/MS)

The content of cannabinoids and ginger compounds was determined on Agilent 1200 HPLC apparatus coupled with an Agilent 6460 JetStream triple quadrupole mass detector (Agilent Technologies, Santa Clara, CA, USA).

For separation of cannabinoids, an Agilent Zorbax Eclipse Plus C18 (100 × 2.1 mm, 2.7 μm) analytical column at 35 °C was used using a mobile phase consisting of water containing 0.1% formic acid (A) and acetonitrile with the addition of 0.1% formic acid (B) with the following elution gradients: 0 min 50% B, 5 min 85% B, 7 min 85% B, 8 min 50% B till 10 min. The flow rate was set at 0.3 mL/min and the injection volume was 5 μL. The mass spectrometer was operated in negative ionization mode with parameters optimized for nitrogen as carrier gas at 300 °C and a flow rate of 6 L/min, the sheath gas temperature was 250 °C, the sheath gas flow was 11 L/min, and the capillary and nozzle voltages were at 3500 kV and 500 kV, respectively.

For analysis of 6-gingerol and 6-shogaol, a Phenomenex Kinetex C18 (150 × 2.1mm, 2.6 μm) analytical column was used. The mobile phase was composed of 0.1% formic acid in water (A) and 0.1% formic acid in acetonitrile (B). The following optimized mobile phase gradient program was used: 0 min 50% B, 3 min 90% B, 6 min 90% B, 7 min 50% B and maintained for 5 min. The flow of the mobile phase was 0.5 mL/min at 30 °C, and 5 µL of sample was injected onto column. Optimized mass spectrometer parameters were gas temperature of 150 °C, gas flow of 5 L/min, nebulizer voltage of 30 V, sheath gas temperature of 300 °C at a flow of 11 l/min, and the capillary and nozzle voltages were 3500 V and 1000 V, respectively.

The multiple reaction monitoring (MRM) mode was applied to detect and quantify ion transitions (Supplementary Materials: Table S1). The quantification of individual components was performed based on the calibration curve methodology.

### 3.8. Gas Chromatography with Mass Spectrometry (GC/MS)

The identification of volatile compounds in the extracts was performed on a Shimadzu GCMS-QP2010 Ultra GC-MS chromatograph (Shimadzu, Duisburg, Germany), using the NIST library, while quantification was performed using the external standard for each individual component. Volatile components from natural extracts were separated on a Phenomenex Zebron ZB5MS chromatographic column, with dimensions of 30 m × 0.25 mm, of 0.25-micron particles with a helium flow rate of 1 mL/min. Separation of aR-turmerone, 1,8-cineole, linalool, and alpha-terpinyl acetate was carried out under the following conditions: temperature gradient: 0–2 min 50 °C, raised to 60 °C at 2 °C/min, after 5 min at 60 °C raised at 10 °C/min to 150 °C, held for 2 min, and finally heated to 240 °C at a rate of 10 °C/min and held for 3 min; the detector and injector were set to a temperature of 250 °C, and the sample injection volume was 1 μL in SPLIT mode at the ratio of 1:10.

### 3.9. Statistical Analysis

Statistical analysis was performed to analyze and assess parameters that influence extraction efficiencies, compare statistical differences between groups, and assess the repeatability of the obtained results. All experiments were performed in triplicate. The R programming language v4.2.2 and Rstudio integrated development environment version 2022.02.3 were used along with packages dplyr [44], ggplot [45], ggpubr [46], PerformanceAnalytics [47], and viridis [48]. For all output parameters, the Shapiro–Wilk test of normality was used to check the distribution of data, and Levene’s test was used to test the homogeneity of variances. Depending on the result, parametric or non-parametric statistical tests were used. For three output variables, namely antioxidant activity and the CBD and CBN content, non-normal distribution was confirmed, while all other variables followed a normal distribution. In the case of normal distribution, the data is reported as mean and standard deviation (SD), while in the case of non-normal distribution, as median and interquartile range (IQR). For two-group comparison, the parametrical *t*-test and non-parametric Mann–Whitney Wilcoxon test were used, while for more than two groups, analysis of variance (ANOVA) and the non-parametric Kruskal–Wallis statistical test were used, with post hoc Tukey and Dunn tests. Spearman’s correlation coefficient was calculated to determine the relationship between variables. *p*-values below 0.05 were defined as statistically significant.

## 4. Conclusions

This study is the first to report the quantities of bioactive components and the synergistic effects when extracting hemp and *Zingiberaceae* plant mixtures and furthermore compares them with extracts from separate materials. Two extraction methods were used, namely ultrasonic-assisted extraction with ethanol and supercritical fluid extraction using CO_2_, for extracting polar and non-polar components, respectively. Obtained extracts were abundant with polar and non-polar antioxidants and phenolic components, as no extraction method was predominant when extracting these compounds. The hemp–cardamom mixture SCE extracts showed the same or even higher levels of total cannabinoids than the individual material. Furthermore, the hemp–ginger mixture SCE extract reached higher levels of CBD than the pure hemp material. Despite the fact that plants of the Zingiberaceae family do not contain cannabinoids, their content in mixtures with hemp was increased in many cases, which indicates a very interesting interaction of molecules. Without the interactions, the expected cannabinoid content should decrease relatively to the amount of plant material added to the cannabis, which was observed for other bioactive components examined in this study. However, in most cases of material mixtures, cannabinoid levels were higher than expected, confirming that simultaneous extraction of hemp with Zingiberaceae plants leads to higher cannabinoid yields. The correlation analysis confirmed the relationship between the components and indicated that the phenolic components were the major source responsible for antioxidant activity in the examined extracts. It also indicated which components were interdependent during extraction, which seemed to be the case for most cannabinoids, 6-gingerol, and 6-shogaol, as well as for alpha-terpinyl acetate and linalool.

## Data Availability

The original contributions presented in the study are included in the article; further inquiries can be directed to the corresponding authors.

## Supplementary Materials

The following supporting information can be downloaded at: https://www.mdpi.com/article/10.3390/molecules28237826/s1, Figure S1: Representative MRM chromatograms obtained for cannabinoid analysis in hemp extract and mixtures: (a) hemp extract, (b) ginger-hemp extract, (c) cardamom-hemp extract, (d) turmeric-hemp extract, and (e) mixture of seven cannabinoid standards (CBDA, CBGA, THCA, CBD, CBC, CBN and THC), Figure S2: Representative MRM chromatograms of 6-gingerol and 6-shogaol (a), ginger extract (b) and ginger-hemp extract (c). MRM transitions are presented in Table S1, Figure S3: Representative GC-MS chromatograms of alpha-terpinyl acetate (a), linalool (b), 1,8-cineol (c), cardamom extract (d), and cardamom-hemp extract (e), Figure S4: Representative GC-MS chromatogram of aR-turmerone (a), turmeric extract (b) and turmeric-hemp (c) extract, Table S1: MS/MS fragmentation (MRM) for all quantified compounds.

## Appendix A

**Table A1:** Antioxidant activity [%] (B). Total phenolic content [mg GA/g extract] of the plant extracts according to the plant material, their mixtures, and extraction method.

Material	Extraction	Antioxidants [%]	IQR AA	Total_Phenols [mg GA/g Extract]	Sd TPC
cardamom	US	0.29	0.03	55.46	4.49
cardamom	SCF	2.99	0.33	43.60	3.00
ginger	US	43.20	3.02	224.16	26.23
ginger	SCF	79.17	4.67	239.35	25.37
hemp	US	6.64	0.65	161.11	6.44
hemp	SCF	6.08	0.18	155.62	17.74
turmeric	US	71.51	6.08	210.69	3.58
turmeric	SCF	6.56	0.49	108.47	13.02
hemp + cardamom	US	9.23	0.36	158.10	16.44
hemp + cardamom	SCF	5.18	0.75	111.48	13.04
hemp + ginger	US	29.50	0.18	235.13	13.64
hemp + ginger	SCF	27.00	0.16	196.51	12.58
hemp + turmeric	US	54.23	4.56	230.56	5.99
hemp + turmeric	SCF	8.23	0.32	145.46	21.82

**Table A2:** The content of cannabinoids in extracts mixtures according to material and extraction method.

Material	Extraction	Mean CBDA	sd CBDA	Median CBD	iqr CBD	Mean CBGA	Sd CBGA	Mean THC	sd THC	Mean THCA	sd THCA	Mean CBC	sd CBC	Median CBN	iqr CBN	Sum of Cannabinoids	sd Cannabinoids
		[mg/g extract]															
hemp	SCF	163.50	17.42	115.17	14.62	11.98	1.11	6.10	0.15	3.43	0.69	2.70	0.21	0.57	0.04	303.43	8.85
hemp	US	167.72	12.01	104.29	3.32	7.51	0.89	5.39	0.21	3.72	0.35	2.18	0.03	0.48	0.03	292.27	15.27
hemp + cardamom	SCF	161.02	2.12	116.31	3.11	13.88	0.35	5.99	0.18	2.62	0.25	2.30	0.03	0.49	0.02	302.60	5.14
hemp + cardamom	US	115.59	6.67	67.24	2.95	4.71	0.32	4.01	0.08	2.79	0.17	1.51	0.02	0.31	0.04	196.16	6.73
hemp + ginger	SCF	83.99	11.09	132.53	4.57	12.11	1.49	5.89	0.05	2.68	0.01	2.28	0.01	0.42	0.06	192,43	50.63
hemp + ginger	US	107.83	14.28	98.70	2.95	7.54	3.39	5.00	0.49	3.93	0.64	1.63	0.01	0.31	0.01	271.99	14.59
hemp + turmeric	SCF	124.17	35.35	65.40	28.69	12.01	0.80	6.54	0,02	3.14	0.49	2.48	0.18	0.54	0.01	214.25	56.72
hemp + turmeric	US	92.96	0.81	48.30	2.59	5.05	0.45	4.22	0.54	2.99	0.14	1.41	0.04	0.29	0.01	155.19	6.34

**Table A3:** The content of 6-gingerol and 6-shogaol in extracts mixtures according to material and extraction method.

Material	Extraction	Mean 6-Gingerol	sd 6-Gingerol	Mean 6-Shogaol	sd 6-Shogaol
				[mg/g extract]	
Ginger	SCF	137.38	7.21	271.12	59.93
Ginger	US	69.51	7.53	172.25	8.24
Hemp + Ginger	SCF	69.98	6.39	122.9	5.78
Hemp + Ginger	US	48.99	8.7	67.96	10.25

**Table A4:** The content of 1,8-cineole, alpha-terpinyl acetate, and linalool in extracts mixtures according to material and extraction method.

Material	Extraction	Mean 1,8-Cineole	sd 1,8-Cineole	Mean Alpha-terpinyl-Acetate	sd Alpha-terpinyl-Acetate	Mean Linalool	sd Linalool
		[mg/g extract]					
Cardamom	SCF	2.65	0.06	485.54	71.82	10.44	0.4
Cardamom	US	9.74	0.33	470.42	24.66	12.23	0.78
Hemp + Cardamom	SCF	8.89	0.86	261.87	9.14	7.18	0.4
Hemp + Cardamom	US	6.57	0.35	204.21	34.21	6.58	0.28

**Table A5:** The content of aromatic-turmerone in extracts mixtures according to material and extraction method.

Material	Extraction	Mean Ar-Turmerone	Sd Ar-Turmerone
		[mg/g extract]	
Turmeric	SCF	147.93	8.15
Turmeric	US	66.01	2.09
Hemp + Turmeric	SCF	50.73	0.52
Hemp + Turmeric	US	32.97	4.22
