# Supplementary material for: Extraction of Active Compounds from Mixtures of Hemp (Cannabis sativa) with Plants of the Zingiberaceae Family
Source: Molecules. 2023 Nov 28;28(23):7826. doi: 10.3390/molecules28237826 (PMC10708002; doi:10.3390/molecules28237826)
Supplement: Supplementary file 1 [file molecules-28-07826-s001.zip › molecules-2649902-supplementary.pdf]

Supplementary Information

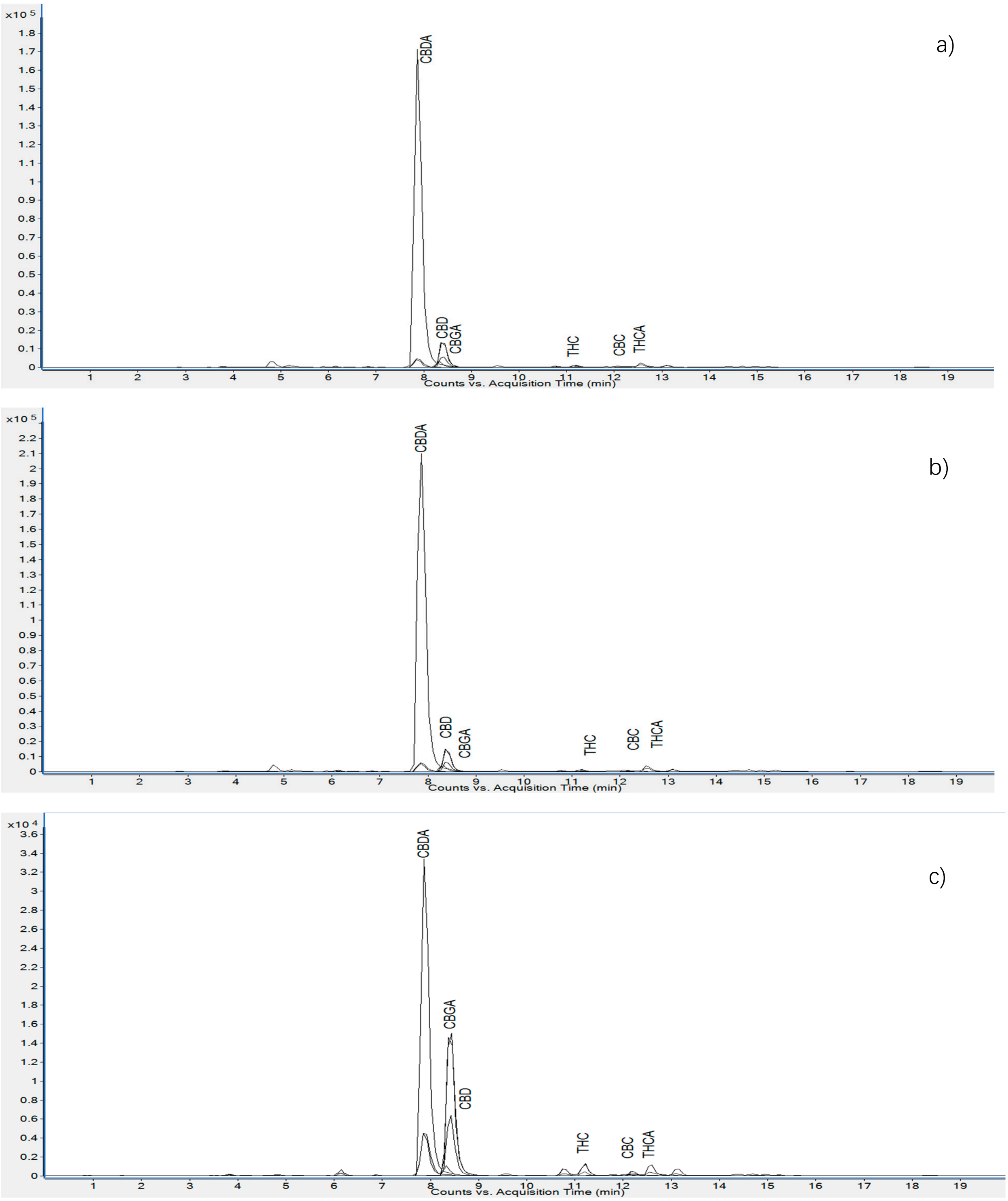

Figure S1: To be continued.

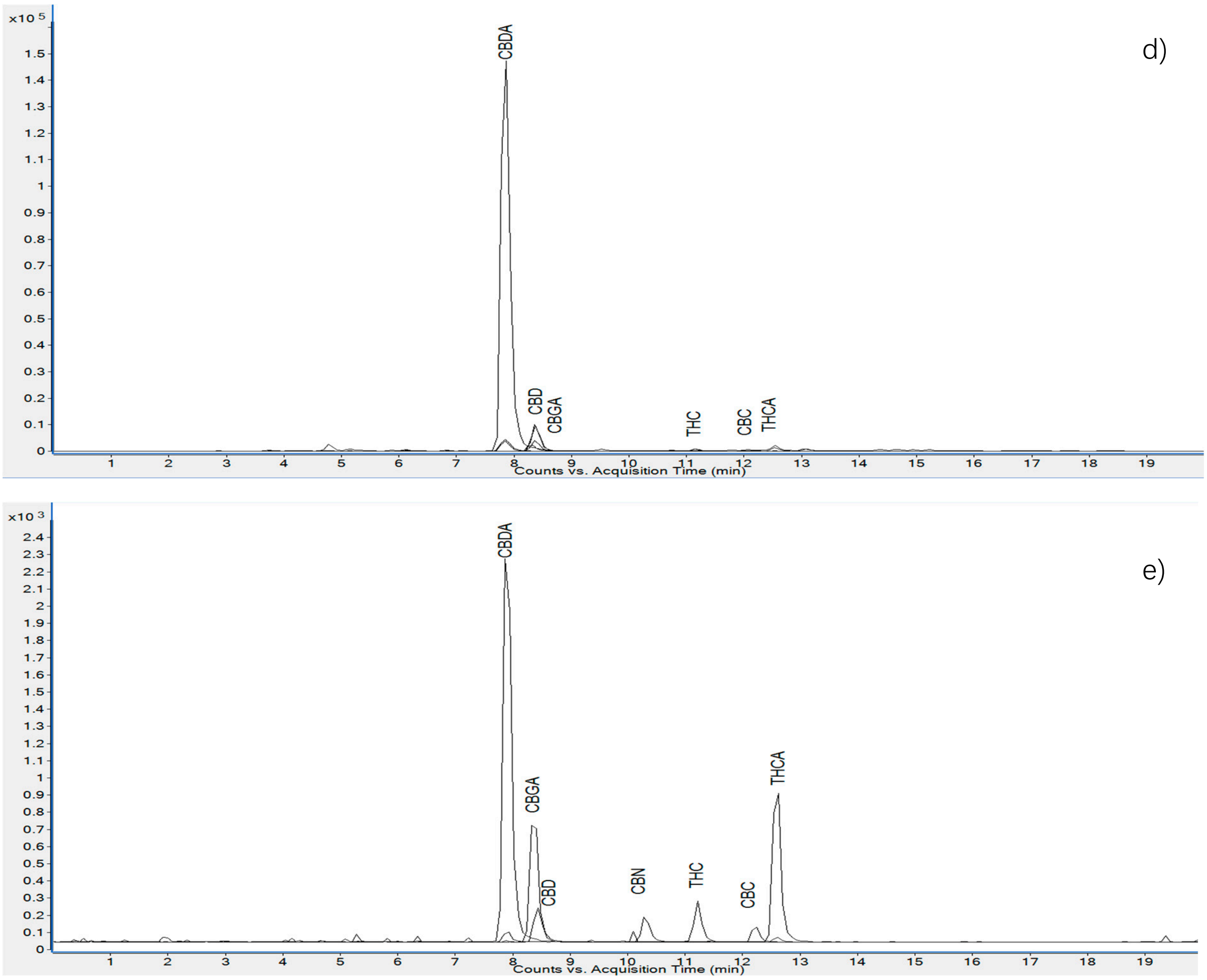

Figure S1: Representative MRM chromatograms obtained for cannabinoid analysis in hemp extract and mixtures: a) hemp extract, b) ginger-hemp extract, c) cardamom-hemp extract, d) turmeric-hemp extract, and e) mixture of seven cannabinoid standards (CBDA, CBGA, THCA, CBD, CBC, CBN and THC)

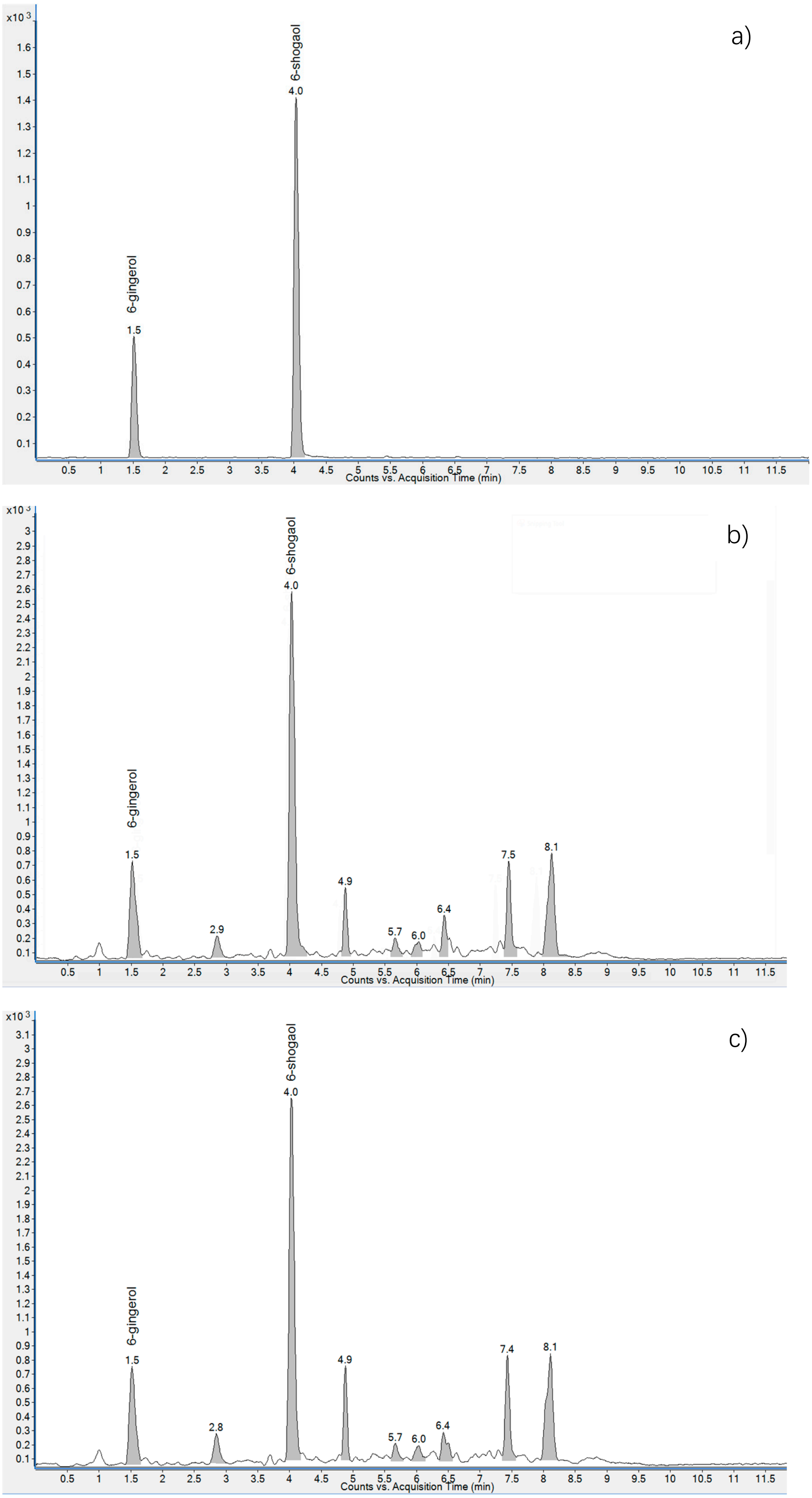

Figure S2: Representative MRM chromatograms of 6-gingerol and 6-shogaol (a), ginger extract (b) and ginger-hemp extract (c). MRM transitions are presented in Table S1.

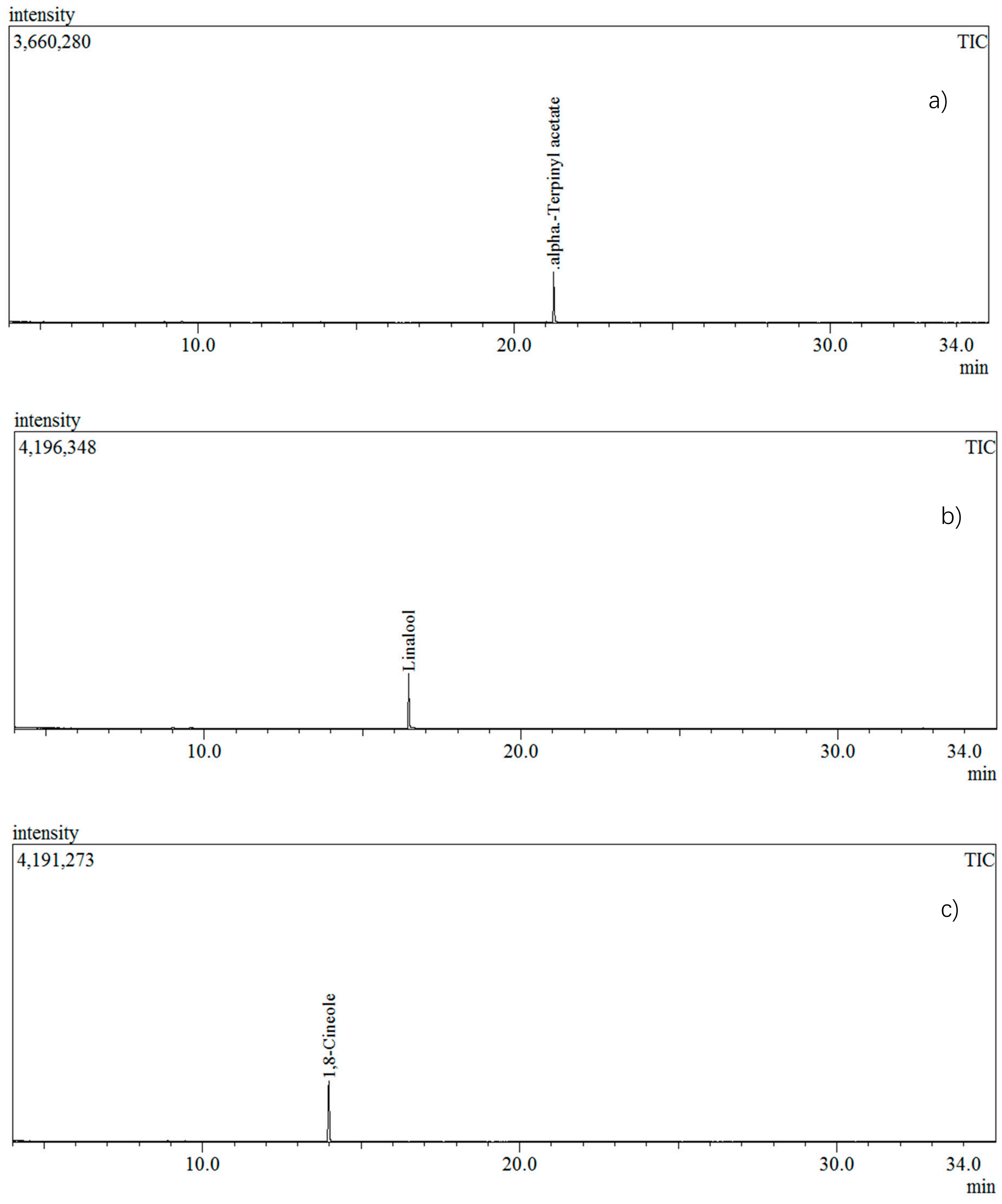

Figure S3: To be continued.

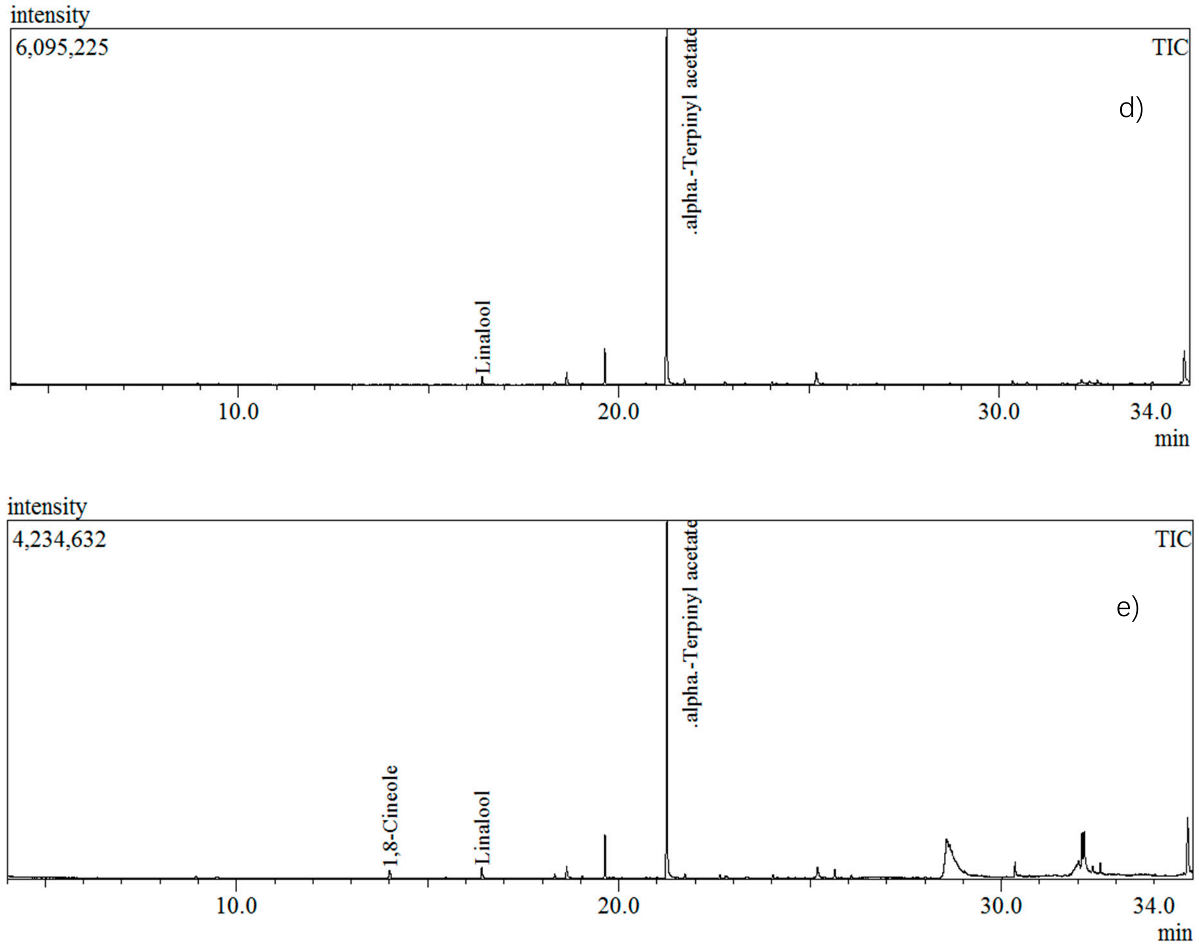

Figure S3: Representative GC-MS chromatograms of alpha-terpinyl acetate (a), linalool (b), 1,8-cineol (c), cardamom extract (d), and cardamom-hemp extract (e).

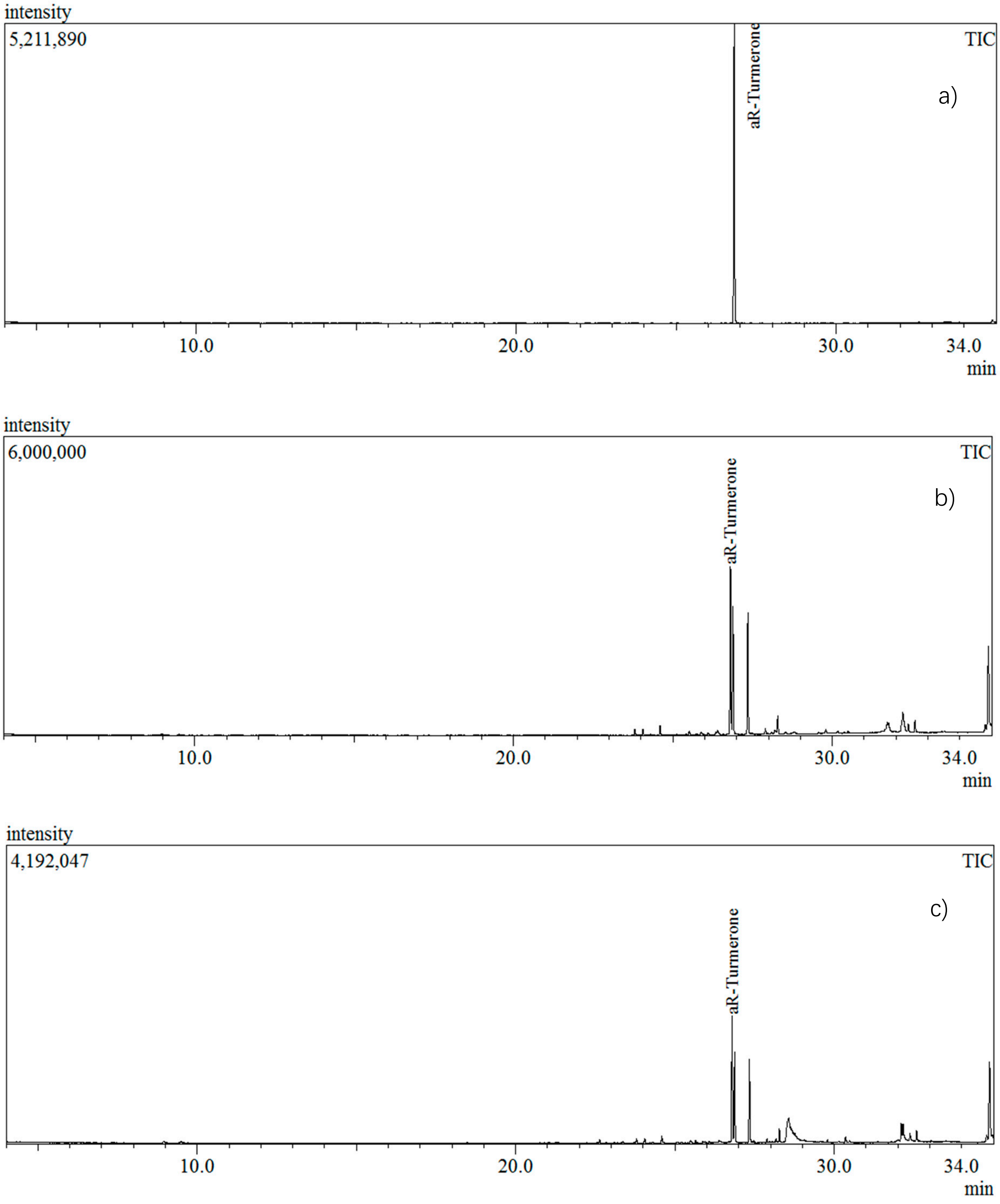

Figure S4: Representative GC-MS chromatogram of aR-turmerone (a), turmeric extract (b) and turmeric-hemp (c) extract.

Table S1: MS/MS fragmentation (MRM) for all quantified compounds.

Analyte	Precursor	Fragment	Collision energy	Polarity
CBGA	361	343, 317	10, 10	negative
CBDA	359	341, 218.8	10, 30	negative
CBD	315.2	193.1, 123.1	20, 36	negative
THCA	357.4	313.1, 245.1	10, 20	negative
THC	315.2	193.1, 123.1	10, 15	negative
CBN	311.2	293.1, 223.1	16, 20	negative
CBC	315.2	259.1, 81.1	12, 15	negative
6-gingerol	317.2	277.2, 177.2	10, 20	positive
6-shogaol	277.2	137.1	15	positive
